# Expression and Methylation of *FGF2*, *TGF-β* and Their Downstream Mediators during Different Developmental Stages of Leg Muscles in Chicken

**DOI:** 10.1371/journal.pone.0079495

**Published:** 2013-11-18

**Authors:** Yue Lu, Sirui Chen, Ning Yang

**Affiliations:** National Engineering Laboratory for Animal Breeding and MOA Key Laboratory of Animal Genetics and Breeding, College of Animal Science and Technology, China Agricultural University, Beijing, China; Harbin Institute of Technology, China

## Abstract

A number of growth factors determine the proliferation of myoblasts and therefore the number of ultimate myofibers. The members of transforming growth factor-beta (TGF-β) family and the fibroblast growth factor 2 (FGF2) have profound effects on skeletal myoblasts proliferation in various animal systems. To investigate their involvement in different stages of avian skeletal muscle development *in vivo*, we detected the mRNA expression and DNA methylation profiles of *TGF-β2*, *TGF-β3, FGF2* and their downstream mediators in leg muscles at embryonic day 10, day of hatch and day 45 posthatch, using both Arbor Acres meat-type and White Leghorn egg-type chickens. By real-time PCR, we found that the expression levels of *TGF-β2*, *TGF-β3*, *Smad3* and *FGF2* were significantly (*P*≤0.01) higher at embryonic day 10, a developmental window of abundant fetal myoblasts expansion, by comparison to day of hatch and day 45 posthatch. The methylation status of the 5′ end region of these four genes was examined subsequently. A section of a CpG island in the 5′ end region of *FGF2* was significantly hypomethylated (*P*≤0.01) at embryonic day 10, compared with neonatal and postnatal stages in both stocks. Our results suggested that *TGF-β2*, *TGF-β3*, *Smad3* and *FGF2* may play important roles in fetal myoblasts proliferation in chicken hindlimb, and the transcription of *FGF2* in this wave of myogenesis could be affected by DNA methylation in 5′ flanking region. These outcomes contribute to our knowledge of the growth factors in avian myogenesis. Further investigation is needed to confirm and fully understand their functions in fetal limb myogenesis in birds.

## Introduction

Meat yield and quality are important factors considered by the poultry industry. Over the past 50 years, the worldwide growing demand for poultry meat has pressured breeders to increase growth rate and muscle mass while reducing abdominal fatness. Currently, chickens are marketed in about half the time and at about twice the body weight compared to 50 years ago [1]. This success is strongly related to a significant increase in the muscle proportion [2]. Ultimate muscle mass is largely determined by hyperplasia and hypertrophy of myofibers [3]. During the process of building muscles (myogenesis), myogenic precursor cells migrate to the appropriate sites of muscle formation followed by their proliferation. The muscle precursors exit the cell cycle and fuse to form myotubes, which develop into multinucleated muscle fibers [4].

Vertebrates undergo three waves of myogenesis (embryonic, fetal and adult) to form the ultimate mature muscle [5]. Embryonic myogenesis generates the first muscle fibers (primary fibers) of the body. Its role is to define the type, shape or location of a muscle, rather than to provide mass. Subsequently, during fetal myogenesis, fetal myoblasts fuse and give rise to secondary fibers (fetal fibers) that surround primary fibers [6]. This wave of fiber formation contributes greatly to the number of fibers that will be found in the mature muscle. During postnatal development, adult myogenesis is involved in muscle regeneration. In the absence of injury, individuals do not form new fibers during the postnatal period and the increase in muscle mass mainly results from an increase in the size of existing fibers (hypertrophy). All of these myogenic events are temporally and spatially regulated by growth factors and transcription factors [7].

Fibroblast growth factor 2 (FGF2) is a member of the fibroblast growth factor (FGF) family, which is one of the major growth factor families in the regulation of muscle growth. FGF2 stimulates skeletal muscle cell proliferation and is a potent inhibitor of differentiation. In the presence of exogenously supplied FGF2, C2C12 myoblasts actively proliferate and show a differentiation-defective phenotype compared with cells cultured in low serum or in the presence of insulin [8]. *FGF2* mRNA and protein are present in proliferating adult mouse MM14 myoblasts but undetectable after terminal differentiation [9]. Murine Sol8 and rat L6 myoblasts also express *FGF2* mRNA, and its expression is down-regulated at the transcriptional level during myogenic differentiation [10]. One mechanism by which FGF2 inhibits muscle gene transcription is through the activation of protein kinase C (PKC) which phosphorylates a conserved site in the basic regions of the myogenic bHLH proteins and thereby prevents their binding to DNA [11]. PKC enzymatic activity is high at the proliferative stage and decreases as myoblasts elongate and fuse. The high level of activity present in undifferentiated myoblasts may be accounted for by PKCα which is one of the conventional isoforms of PKC [12].

Several members of the transforming growth factor-beta (TGF-β) family play important roles in regulating muscle growth and atrophy [13]. The TGF-β superfamily consists of over 50 structurally related ligands, which fall into three major subfamilies: TGF-β, bone morphogenic protein (BMP), and activin [14]. TGF-β inhibits differentiation of fetal, but not embryonic myoblasts. The treatment of mouse limb bud organ cultures with TGF-β neutralizing antibodies results in the premature appearance of large myotubes [15]. Furthermore, three mammalian isoforms of TGF-β (TGF-β1, TGF-β2 and TGF-β3) promote proliferation and delay differentiation of C2C12 myoblasts in an isoform-independent manner [16]. Smad family member 3 (Smad3) is the key mediator of TGF-β inhibition of muscle differentiation by suppression of the transactivation properties of MyoD and MEF2C [17]. In mouse, *Smad3*-null myoblasts exhibit impaired proliferation, differentiation and fusion, resulting in the formation of atrophied myotubes. *Smad3*-null satellite cells show a reduced propensity for self-renewal, which may lead to a progressive loss of satellite cell number [18].

During development, *TGF-β* and *FGF* genes activation is regulated to restrict their expression to the correct cell type and developmental stage. The spatially and temporally restricted pattern of gene expression implies that these genes might play important roles in the corresponding physiological processes. Genes displaying altered expression levels could have undergone an increase or decrease in DNA methylation. In mammals, DNA methylation occurs mostly at the CpG dinucleotides except for the CpG sites in CpG islands which are relatively lowly methylated [19], [20]. The methylation of 5′ end of gene is usually suppressive for gene expression. As for myogenesis, Heidari *et al*. (2001) reported that during the mouse G8 myoblast differentiation the genome-wide CpG sites were demethylated first, followed by a gradual remethylation. The expression of muscle-related genes also changed. The down-regulated genes were those involved in DNA synthesis and replication (e.g., *G/T mismatch DNA glycosylase*, *High mobility group box 2 a*nd *Cell division cycle 2 homolog A*) and the up-regulated genes were those which are muscle specific (e.g., *Troponin-T3*, *Actin-alpha1* and *Myogenin*). The *de novo* methylation might be responsible for the down-regulated muscle-related genes [21]. In the chicken genome, a total of 20,224 CpG islands were identified using the criteria of length >200 bp, G+C content >50% and CpG observed to expected >0.6 [22]. About 10% of the methylated CpG islands are in promoter regions with about 7.3% methylated in Arbor Acres broiler muscle and 13.1% in Red Jungle Fowl (RJF) muscle [23].

Although much is known regarding the expression and roles of the members in TGF-β and FGF signaling cascades in myogenesis, very little information exists *in vivo* detailing the expression and methylation patterns of these members in avian skeletal myofiber hyperplasia and hypertrophy. In the current study, we designed to quantify *TGF-β2*, *TGF-β3, FGF2* mRNA expression and their downstream mediators (*Smad3* and *PKCα*), and evaluated the DNA methylation status of the 5′ upstream regions of these genes in the lower leg muscles of fetal, newly hatched and adult chicks. The results suggest that *TGF-β2*, *TGF-β3, FGF2* and *Smad3* play important roles in the regulation of fetal myoblast proliferation in the chicken hindlimb and the transcription of *FGF2* during fetal myogenesis may be affected by DNA methylation.

## Materials and Methods

### Ethics Statement

Animal experiments were approved by the Animal Care and Use Committee of China Agricultural University and the experiment was performed according to regulations and guidelines established by this committee.

### Experimental Animals and Sample Collection

Fertilized eggs of meat-type broilers (commercial Arbor Acres (AA)) and egg-type layers (an experimental line of White Leghorn (WL) which has been selected for egg production for over 10 years in the experimental station of China Agricultural University) were obtained from China Agricultural University and were incubated in the same incubator at the same time. The chickens were reared individually since hatch with the same food and water supply system to minimize the influence of the environment. From hatch to 28 days of age, birds received starter feed with 21% protein and 2,910 kcal of ME/kg, and then from 29 to 45 days of age, a diet with 20% CP and 3,010 kcal of ME/kg was provided. Lower leg muscles were isolated from four randomly selected males from each stock at embryonic day 10 (E10), day of hatch (D1) and day 45 posthatch (D45), respectively. These times were chosen to represent the stages that the fetal myoblasts are most abundant (E8 to E12), myofiber number is becoming fixed (before or shortly after hatching), and broilers are selected for breeding (D42 to D49) [24], [25]. The sex of fetuses at E10 was molecularly identified by amplification of the *CHD1* gene [26]. Tissues were quickly stored in RNA fixer (BioTeke Co., Ltd, Beijing, China), preserved at 4°C overnight and transferred to −80°C until analysis.

### Quantitative Real-time PCR

Total RNA was isolated from lower leg muscles using Trizol reagent (Invitrogen) according to the manufacturer’s instructions and dissolved in RNase-free water. The first strand cDNA was synthesized from total RNA using M-MLV reverse transcriptase (Promega Biotech Co. Ltd., Beijing, China).

To avoid amplification of contaminating genomic DNA, the primers specific for *TGFβ-2*, *TGFβ-3*, *FGF2*, *Smad3* and *PKCα* were designed to span exon and intron boundaries. Primers were designed using known nucleotide sequences from GenBank database and Primer Express software (Version 3.0, Applied Biosystems, USA) (Table 1).

**Table 1:** Primers used in the real-time PCR analysis.

Genes	GenBank Accession Number	Sequence	Product (bp)
*GAPDH*	NM_204305.1	F: 5′- CTTTCCGTGTGCCAACCC -3′	108
		R: 5′- CATCAGCAGCAGCCTTCACTAC -3′	
*TGF-β2*	XM_003640970.2	F: 5′- TATCATCACCAGGACAGCGT -3′	177
		R: 5′- TATTGGAAGGCACAAAGGTG -3′	
*TGF-β3*	NM_205454.1	F: 5′- TCTTTACATTGACTTCCGAC -3′	237
		R: 5′- TCCTCCCAACATAGTACAAG -3′	
*Smad3*	NM_204475.1	F: 5′- GCTCTCTTGGCTCAGTCTGT -3′	195
		R: 5′- CATCTGTGTGAGGACCTTGT -3′	
*FGF2*	NM_205433.1	F: 5′-AGAGGAGTAGTATCAATCAAAG -3′	174
		R: 5′- TGCCACATACCAATCAGAGT -3′	
*PKCα*	NM_001012804.1	F: 5′- ATCCAAAATCGCTGTCCAAAG-3′	114
		R: 5′- CGTGTTCCCTGATGTCTCTT -3′	

Real-time PCR of gene expression was conducted using standard curves. For construction of standard curves of target and reference genes, PCR products of reference gene (*GAPDH*) and target genes were cloned into pMD^TM^19-T Vector (Takara Bio Inc., Japan) and 10-fold serial dilutions (10^−1^ to 10^−8^) of the relevant plasmid DNA preparations were made and assayed in duplicate. Each cDNA sample was then analyzed by triplicate real-time PCRs using ABI Prism 7300 system (Foster City, CA). PCR was run along with a dilution series of the standard that served as the calibrator. A no template control was included with each run. The real-time PCR reactions were performed in a final volume of 15 µl with 1 µl of cDNA, 100 nM of each gene-specific primer and 1×PCR mix (Power SYBR® Green PCR Master Mix, Applied Biosystems). The optimum thermal cycling parameters included 95°C for 10 min, 40 cycles of 95°C for 15 s, 60°C for 1 min, 95°C for 15 s, 60°C for 30 s and 95°C for 15 s.

### DNA Methylation Analysis

The 5′ end of the gene, the putative proximal promoter regions of the target genes, is important for the regulation of gene expression. Most of the promoter regions are associated with CpG islands and are lowly methylated. The methylation of these regions is usually suppressive for gene expression [23]. Accordingly, we focused on the CpG islands in 2000 bp sequences upstream of the transcription start site (here we assumed it begins where exon1 sequence starts) of target genes. The CpG islands were evaluated by CpGplot (http://www.ebi.ac.uk/Tools/emboss/cpgplot/).

Genomic DNA was extracted from lower leg muscles using a traditional phenol-chloroform protocol. The quality and quantified were evaluated by gel electrophoresis and a NanoDrop spectrophotometer (GE Healthcare Life Sciences, Uppsala, Sweden). A total of 2 µl of genomic DNA from each sample was treated with sodium bisulfite using an EZ DNA methylation kit (Zymo Research, Orange, CA), and the modified DNA was amplified by PCR. The target regions were amplified using the primer pairs that were designed with Sequenom online EpiDesigner (http://epidesigner.com). Each forward primer was tagged with a decamer (5′-AGGAAGAGAG-3′), and each reverse primer had a T7-promoter tag (5′-CAGTAATACGACTCACTATAGGGAGAAGGCT-3′) for transcription *in vitro*. The amplicon primers for the CpG islands are detailed in Table 2. The sequence and chromosomal location of all amplicons are shown in Figure S1, S2, S3 and S4. Sequenom MassARRAY platform (CapitalBio, Beijing, China) was used to perform the quantitative methylation analysis. This system uses matrix-assisted laser desorption/ionization time-of-flight (MALDI-TOF) mass spectrometry in combination with base-specific cleavage. Quantitative results for each CpG site or an aggregate of multiple CpG sites were generated by Epityper software version 1.0 (Sequenom).

**Table 2:** Primers used in the DNA methylation analysis.

Gene	Amplicon^1^	Direction	Sequence	No. ofCpG site^2^	Product(bp)
*TGF-β2*	**TGF-β2-amp**	Forward	5′-aggaagagagGAGAGGGAGATTTATAAGGGTTTTT-3′	33	399
		Reverse	3′-cagtaatacgactcactatagggagaaggctCTACTTTCCCCTAAAATTACCCCA- 5′		
*TGF-β3*	**TGF-β3-amp1**	Forward	5′-aggaagagagATGTGGAAGGGATTTTTAAAGGTTA- 3′	4	363
		Reverse	3′-cagtaatacgactcactatagggagaaggctAATTTCCTACATTTACCAAAACAACC- 5′		
	TGF-β3-amp2	Forward	5′-aggaagagagGGGAATGGGTTTTATAAGGAGTAAA- 3′	7	378
		Reverse	3′-cagtaatacgactcactatagggagaaggctACCTTTTAAACAAATACCCAACTCA- 5′		
*Smad3*	Smad3-amp1	Forward	5′-aggaagagagTTGATTGAGTGGTGTTTAGTTGTGT- 3′	40	431
		Reverse	3′-cagtaatacgactcactatagggagaaggctCATTCCCAATCTAACTCTCATACAAA- 5′		
	Smad3-amp2	Forward	5′-aggaagagagAGTTAGATTGGGAATGAGTTGGAGT- 3′	41	337
		Reverse	3′-cagtaatacgactcactatagggagaaggctCCTTTCTTCCAACCCAAAAAAC- 5′		
*FGF2*	FGF2-amp1	Forward	5′-aggaagagagTTGGGAAGTAAGAAGTAAGAGTTGG- 3′	19	449
		Reverse	3′-cagtaatacgactcactatagggagaaggctACTTTCCTAATTCCAAAACAACACA- 5′		
	**FGF2-amp2**	Forward	5′-aggaagagagTTGTTTTGGAATTAGGAAAGTTTTTT- 3′	9	233
		Reverse	3′-cagtaatacgactcactatagggagaaggctAACACCTATTACCTCCAACAACAAC- 5′		

1 Bold symbol represents the amplicons with good quality.

2 No. of CpG site = the number of CpG sites in each amplicon.

### Statistical Analysis

The expression of target genes was normalized against the expression of *GAPDH* gene and expressed as ratios. MassARRAY has a detection spectrum range from 1,700 to 7,500 Da. For the quality control of Epityper data, the low coverage CpG sites across the sample and across the CpG units (coverage ≤50%) were cut off.

The RNA expression and DNA methylation variation among different time points within each stock and variation between two stocks per time point were analyzed by SAS 8.0 (SAS Institute Inc., Cary, NC) using one-way ANOVA and Student’s t-Test, respectively. Comparisons were considered significant at *P*≤0.05 and highly significant at *P*≤0.01.

## Results and Discussion

### *TGF-βs, Smad3* and *FGF2* were Highly Expressed during Fetal Development of Leg Muscles

**Figure 1:**
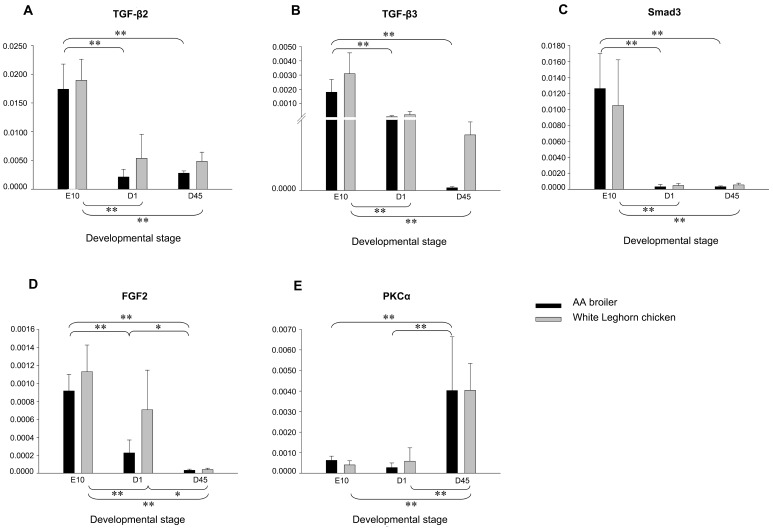
Expressions of candidate genes in leg muscles of AA broiler and White Leghorn chicken. Panels A, B, C, D and E show *TGF-β2*, *TGF-β3*, *Smad3*, *FGF2* and *PKCα* mRNA expressions in lower leg muscles, respectively. Groups are as follows: AA broiler and White Leghorn chicken, sampled at embryonic day 10, day of hatch and day 45 posthatch. Target gene expression is presented relative to *GAPDH* expression and normalized to a calibrator. Error bars represent standard deviation of the means. *P≤0.05, **P≤0.01.

Expression of *TGF-β2*, *TGF-β3*, *Smad3*, *FGF2* and *PKCα* mRNA in hindlimb muscles of AA broilers and WL chickens, at E10, D1 and D45 was quantified (Figure 1). There was a gradual decrease in expression of *TGF-β2*, *TGF-β2* and *Smad3* mRNA with the animal age in both stocks. The expressions of these three genes were significantly (*P*≤0.01) higher at E10 than those at D1 and D45. No significant difference was detected between the two stocks at any time point. Proliferation and differentiation of myoblasts are mutually exclusive. Failure to exit the cell cycle prevents myoblast differentiation [27]. TGF-β inhibits cell line and primary cultures of rat and chicken embryo myoblasts from fusing into multinucleated myotubes [28]. A decrease in TGF-β signaling can improve the success of myogenic differentiation of either endogenous (local or systemic) or exogenous (transplanted) stem cell populations [16]. Smad3 is the key mediator of TGF-β inhibition of myogenesis [29]. It is critical for TGF-β signaling in myoblasts that Smad3 physically interacts with MyoD and MEF2C [17]. MicroRNAs are also implicated in the genetic regulation of Smad3 dependant TGF-β signaling during skeletal muscle differentiation. MiR-24 is shown up-regulated during C2C12 myoblast differentiation and enhances myogenic differentiation, and TGF-β1 represses miR-24 transcription on the presence of Smad3 and a Smads binding site in the promoter region of miR-24 [30]. Together with our results, elevated Smad3 dependant TGF-β signaling may play important roles in sustaining fetal myoblast proliferation in chicken hindlimb muscles.

Similarly, in both AA and WL chickens, *FGF2* was expressed significantly (*P*≤0.01) higher at E10 than at D1 and D45. In addition, its expression was significantly (*P*≤0.05) higher at D1 than that at D45. However, the expression of *PKCα* showed a reverse trend with significantly lower (*P*≤0.01) transcript abundance at E10 than at D1 and D45. No significant difference was found between the two stocks at any specific time point. FGF2 is a potent stimulator of myoblast and satellite cell proliferation, and an effective inhibitor of differentiation [31]. The expression of *FGF2* mRNA was higher during embryonic development of a growth-selected turkey line compared with a random-bred control line. Increased *FGF2* expression during the hyperplastic period of muscle growth would result in the formation of more muscle fibers prior to hatch [32]. FGF2 maintains the skeletal muscle cells in a state of proliferation by inhibiting the transcription of Myogenin, a muscle specific transcriptional regulatory factor required for the initiation of myotube formation [33]. Our results showed that *FGF2* was highly transcribed in leg muscles during the stage when the fetal myoblasts are most abundant, which suggests that *FGF2* may stimulate fetal myoblast proliferation in chicken.

FGF2 suppresses Myogenin function by inducing phosphorylation of a conserved site in the DNA-binding domain. PKC can substitute for FGF2 to phosphorylate this domain both in vivo and in vitro and mediates repression of Myogenin activity [34]. In myoblasts obtained from chicken breast muscles, the expressions of PKC isoforms show high level of PKCα in the proliferating phase that markedly decreases as myoblasts differentiate. Thus, this isozyme may have an important role in maintaining myoblast proliferation [12]. Nevertheless, in this study, *PKCα* mRNA amount was significantly (*P*≤0.01) lower during fetal myogenesis compared to neonatal and postnatal developmental stages, suggesting that, in chicken, the expression and role of *PKCα* might be tissue-specific during fetal myogenesis, and it may participate in hypertrophy of myofibers in developing lower leg muscles.

### Promoter DNA Methylation and mRNA Expression Pattern of FGF2 were Compatible

Genes displaying altered expression levels could have undergone an increase or decrease of methylation. Those affected by methylation changes would be expected to have some methylated CpG sites within proximal promoter regions. Hence, we investigated the methylation status of 5′ ends of *FGF2*, *TGF-β2*, *TGF-β3* and *Smad3* because of their significantly (*P*≤0.01) higher transcription at E10 compared to D1 and D45. CpGplot identified two CpG islands in *FGF2* and *Smad3*, and one island in *TGF-β2*, respectively. Since there was no CpG island found in *TGF-β3*, 700 bp sequences (TGF-β3-sqe) upstream of the transcription start site of this gene was selected directly for methylation detection. The amplicons for the selected CpG islands were FGF2-amp1 and FGF2-amp2 in *FGF2*, Smad3-amp1 and Smad3-amp2 in *Smad3* and TGF-β2-amp in *TGF-β2*. Additionally, two amplicons, TGF-β3-amp1 and TGF-β3-amp2, were performed for TGF-β3-sqe (Table 2). TGFβ-2-amp and FGF2-amp2 covered part of their corresponding CpG islands because of unknown DNA sequences downstream of the islands. According to the quality control of Epityper data (the coverage of the CpG sites across the sample and across the CpG units ≥50%), the methylation detection showed the amplicons FGF2-amp2, TGF-β3-amp1 and TGF-β2-amp were with good quality and they were used for further analysis.

**Figure 2:**
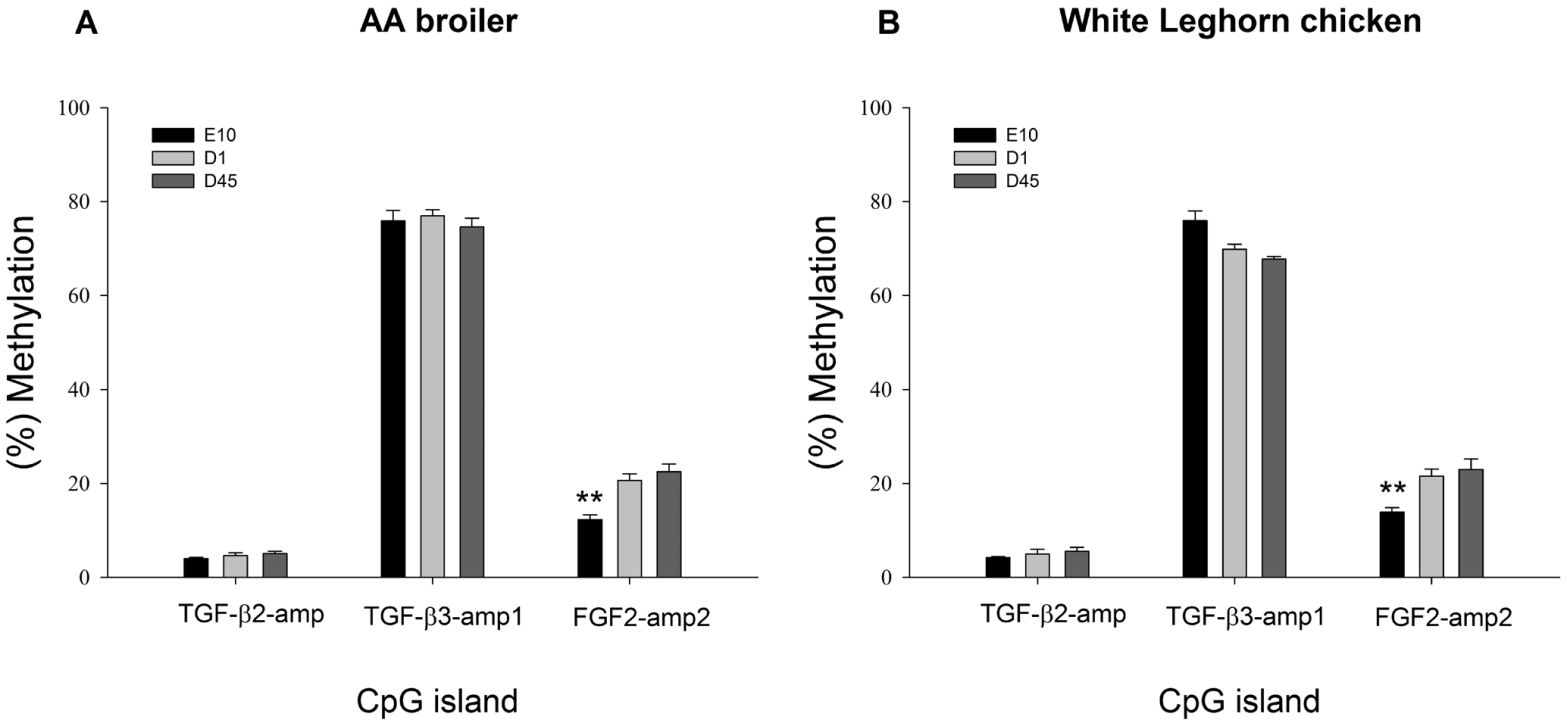
DNA methylation of candidate genes in leg muscles of AA broiler and White Leghorn chicken. Panels A and B show DNA methylation of TGF-β2-amp, TGF-β3-amp1 and FGF2-amp2 in lower leg muscles of AA broiler and White Leghorn chicken, respectively. (%) methylation: the number of methylated CpG sites/(the number of methylated CpG sites + the number of unmethylated CpG sites). Error bars represent standard error of the means. *P≤0.05, **P≤0.01.

**Figure 3:**
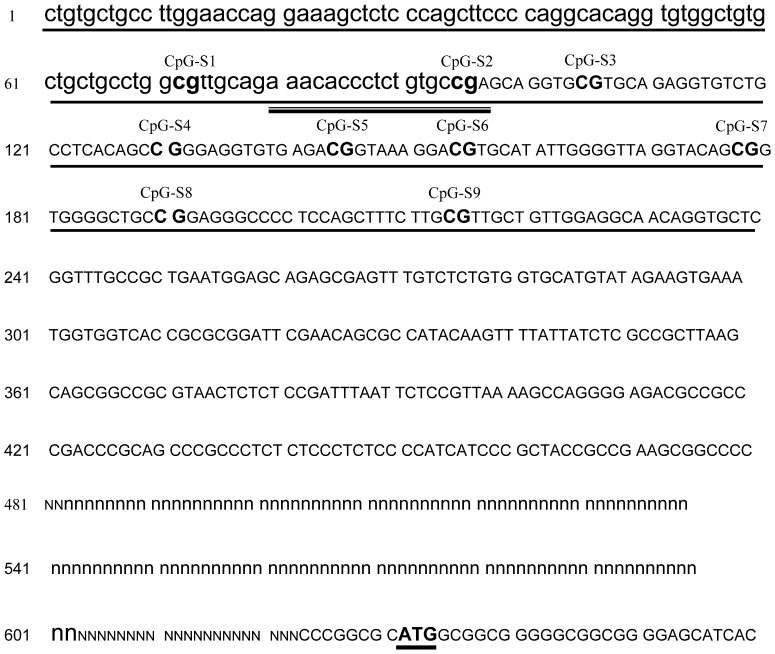
Map of FGF2-amp2 and the CpG sites in this amplicon. The CpG island in proximity of initiation codon (ATG) is indicated as capital letters. Amplicon FGF2-amp2 was underlined with solid line. Individual CpG sites in FGF2-amp2 are indicated as bold type and numbered from CpG-S1 to CpG-S9. Pax3 binding site was underlined with hollow line. The initiation codon (ATG) is also underlined. N and n represent the unknown sequence.

**Figure 4:**
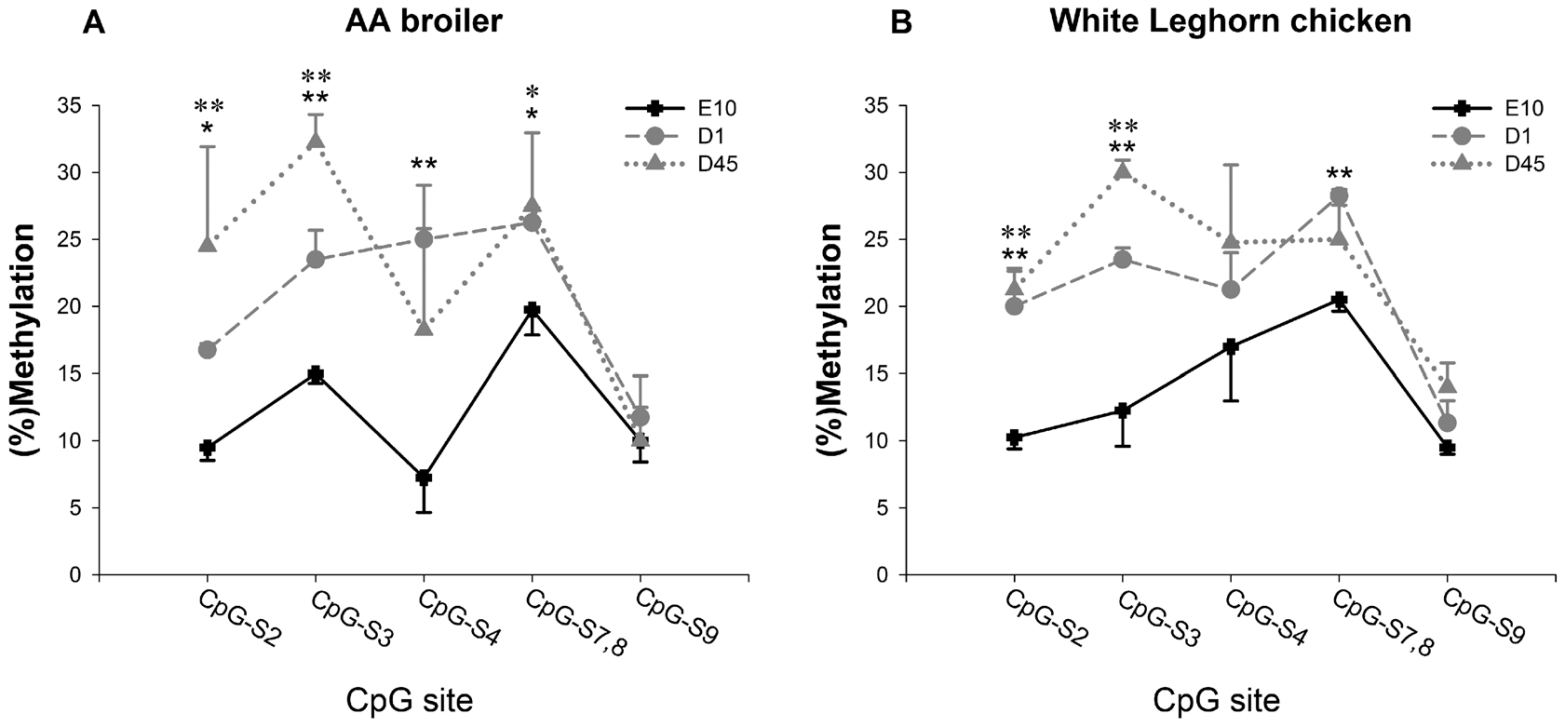
Site-specific CpG methylation in FGF2-amp2 in leg muscles of AA broilers and White Leghorn chickens. Panels A and B show DNA methylations of CpG-S2, CpG-S3, CpG-S4, CpG-S7,8 and CpG-S9 in FGF2-amp2 in lower leg muscles of AA broilers and White Leghorn chickens, respectively. (%) methylation: the number of methylated CpG sites/(the number of methylated CpG sites + the number of unmethylated CpG sites). Error bars represent standard error of the means. *The difference between E10 and D1 is significant (*P*≤0.05), **The difference between E10 and D1 is highly significant (*P*≤0.01) *The difference between E10 and D45 is significant (*P*≤0.05), **The difference between E10 and D45 is highly significant (*P*≤0.01).

As shown in Figure 2, among these three CpG amplicons, FGF2-amp2 contained significantly (*P*≤0.01) lower methylation level at E10 than at D1 and D45, in both AA broiler and WL chickens. However, there was no significant difference between the two stocks at any time point. The amplicon for FGF2-amp2 covered the front section of the corresponding CpG island (Figure 3). Six out of nine covered CpG sites over this amplicons were analyzed. The methylation levels of these six CpG sites were also evaluated separately. As shown in Figure 4, although methylation levels varied at the different CpG sites, the prenatal methylation levels were all lower than the neonatal and postnatal levels. Significant differences (*P*≤0.05 or *P*≤0.01) were found at CpG-S2, CpG-S3, CpG-S4 and CpG-S7, 8 in AA broilers and CpG-S2, CpG-S3 and CpG-S7, 8 in WL chickens, respectively. According to the mRNA expression analysis, fetal expression level of *FGF2* mRNA was significantly higher (*P*≤0.01) than neonatal and postnatal levels in both stocks. It indicated that the stimulation of fetal myoblast proliferation by *FGF2* might be affected by DNA methylation in 5′ end region of this gene. Through DNA methylation, it is possible to change the information content of DNA that can affect differentiation and development [35]. One way in which DNA methylation represses transcription is by blocking the binding of transcription factors and promoting the formation of an inactive compact chromatin structure which is recognized as repressive signal for gene transcription [36], [37]. We identified transcription factor binding sites (TFBS) in FGF2-amp2 using MATINSPECTOR [38]. CpG–S2, which showed significant (*P*≤0.01 or *P*≤0.05) lower methylation at E10 compared with D1 and D45 in both stocks, was located in a *cis* element for Pax3 DNA binding (Figure 3). The role of Pax3 in different aspects of myogenesis has been widely studied. It is required for dermomyotome formation, activation of *MyoD* and *Myf5* expression and limb musculature development [39]. A population of muscle progenitors has been identified that expresses both *Pax3* and *Pax7*, but no additional markers of skeletal muscle such as *MyoD* or *Desmin*. These myogenic precursor cells proliferate in embryonic and fetal muscles of the trunk and limbs throughout development and determine the formation of the fetal muscles [40]. Furthermore, Lagha *et al*. (2008) have shown that Pax3 acts genetically upstream of FGF signaling components (*FGFR4* and *Sprouty1*), which in turn also regulate the balance between the size of the proliferating myoblast progenitor pool and differentiating muscle [41]. According to our results, we speculated that Pax3 may be also required for the activation of *FGF2* in fetal myoblasts of chicken hindlimb.

In conclusion, our results showed that *TGF-β2*, *TGF-β3*, *Smad3* and *FGF2* exhibited significantly (*P*≤0.01) higher transcripts at the fetal developmental stage compared with neonatal and postnatal stages in lower leg muscles, which suggested that these genes may play important roles during fetal myogenesis in chicken hindlimb. Furthermore, a section of a CpG island in *FGF2* 5′ region was significantly (*P*≤0.01) hypomethylated at fetal stage, suggesting that the transcription of *FGF2* in proliferating fetal myoblasts could be affected by DNA methylation. Further work is required to validate the involvements of these growth factors in avian fetal limb myogenesis and elucidate the mechanisms underlying this association.

## Supporting Information

Figure S1 **TGF-β2 schematic representation of genomic regions studied via Sequenom MassARRAY platform.** The exon 1 of the transcript is shown as gray box. The 2000 bp sequence upstream of exon 1 is shown as bold line. For the amplicon reported in the black frame, CpG sites are highlighted in red, and the sequences located in CpG islands are additionally indicated as capital letters.
(TIF)

Figure S2 **TGF-β3 schematic representation of genomic regions studied via Sequenom MassARRAY platform.** The exon 1 of the transcript is shown as gray box. The 2000 bp sequence upstream of exon 1 is shown as bold line. For each amplicon reported in the black frames, CpG sites are highlighted in red.
(TIF)

Figure S3 **Smad3 schematic representation of genomic regions studied via Sequenom MassARRAY platform.** The exon 1 of the transcript is shown as gray box. The 2000 bp sequence upstream of exon 1 is shown as bold line. For each amplicon reported in the black frames, CpG sites are highlighted in red, and the sequences located in CpG islands are additionally indicated as capital letters.
(TIF)

Figure S4 **FGF2 schematic representation of genomic regions studied via Sequenom MassARRAY platform.** The exon 1 of the transcript is shown as gray box. The 2000 bp sequence upstream of exon 1 is shown as bold line. For each amplicon reported in the black frames, CpG sites are highlighted in red, and the sequences located in CpG islands are additionally indicated as capital letters.
(TIF)
